# Symmetry-Guided Functional Pathways of Intercalation-Free Rhombohedral (*R3*) Hafnia Derived from the Fluorite Phase for Low-Coercive Ferroelectric Memory

**DOI:** 10.1021/acsami.5c23427

**Published:** 2026-04-01

**Authors:** Mochamad Januar, Cheng-Hong Liu, Abhijit Aich, Jia-Yang Lee, Siddheswar Maikap, Min-Hung Lee

**Affiliations:** † Program for Semiconductor Devices, Materials, and Hetero-integration, Graduate School of Advanced Technology, 33561 National Taiwan University, Taipei 106319, Taiwan; ‡ Thin Film Nano Tech. Lab., Department of Electronics Engineering, 56081 Chang Gung University, Taoyuan 33302, Taiwan; § Department of Obstetrics and Gynecology, Keelung Chang Gung Memorial Hospital, Keelung 204, Taiwan; ∥ Graduate Institute of Electronics Engineering, National Taiwan University, Taipei 106319, Taiwan; ⊥ Institute of Applied Mechanics, National Taiwan University, Taipei 106319, Taiwan

**Keywords:** Rhombohedral hafnia, intercalation-free rhombohedral, *R3* phase, intrinsic ferroelectricity, symmetry-driven polarization, low-coercive field, phase-selective HRTEM

## Abstract

Rhombohedral hafnia-based ferroelectrics promise low-coercive, scalable nonvolatile memories, yet their realization has traditionally relied on complex cation intercalation or external stress. Here, we demonstrate a possible intrinsic route to the rhombohedral (*R3*) phase in Hf_1–*x*_Zr_*x*_O_2_ through symmetry breaking of the parent fluorite lattice. First-principles calculations under *R3* symmetry-constrained equation-of-state conditions show that the 12-atom fluorite-derived configuration, at equiatomic composition (*x* = 0.5), stabilizes an intrinsically polar *R3* ground state with spontaneous polarization *P*_*s*_ = 44.9 μC cm^–2^, dielectric permittivity *ε*_*r*_ = 51.3, an ultralow switching barrier of 27.8 meV f.u^.–1^, and a coercive field of 0.46 MV cm^–1^. Distinct from orthorhombic *Pca2*_*1*_, the *R3* structure shows nonmonotonic dielectric behavior, revealing a symmetry-renormalized polarization mechanism beyond conventional Vegard-type ferroelectricity. Moreover, the *R3* phase stabilizes at reduced thickness, with low built-in potential at proper electrodes preserving its low coercive field. Experiments using fast Fourier transform and geometric-phase analysis validate these predictions, and *R3*-phase-dominant Hf_1–*x*_Zr_*x*_O_2_ capacitors exhibit comparably low coercive fields (0.65 MV cm^–1^) and enhanced dielectric permittivity *ε*_*r*_ = 39.1.

## Graphical abstract

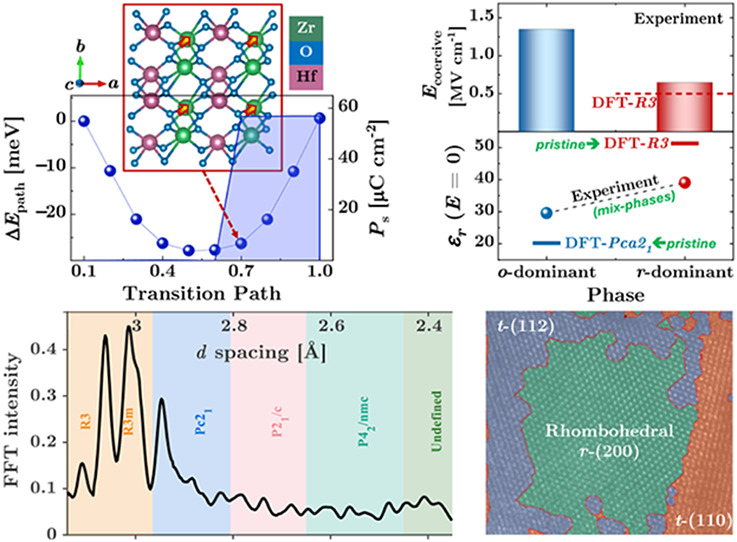

## 1 Introduction

In the pursuit of next-generation nonvolatile memory technologies, the discovery of ferroelectricity in hafnium oxide (HfO_2_) has been a genuine game changer, propelling ferroelectric devices to the forefront of nanoelectronics.^1,2^ Unlike conventional perovskite ferroelectrics such as lead zirconate titanate (PZT), which suffer from poor scalability and integration challenges, HfO_2_ thin films can sustain robust polarization down to the nanometer scale while integrating seamlessly with silicon CMOS processes.^3,4^ This unique advantage has reshaped the landscape of memory technologies, fueling renewed interest in ferroelectric field-effect transistors (FeFETs) and ferroelectric random-access memory (FeRAM) based on HfO_2_.^1,5,6^ Indeed, HfO_2_-based ferroelectrics combine the desired scalability of a simple fluorite lattice with compatibility to standard semiconductor fabrication, addressing key limitations of perovskite counterparts.^3,7–9^ However, a major hurdle for HfO_2_ devices has been their relatively high coercive field, which translates to high operating voltages and causes reliability concerns.^4,10–12^ Reducing this coercive field—without sacrificing polarization or endurance—remains a critical step toward practical ultradense memory implementation.

The ferroelectricity in HfO_2_ is commonly associated with a metastable orthorhombic (*o*)-phase (space group *Pca2*_*1*_), which serves as the canonical polar structure in doped HfO_2_ and Hf_1–*x*_Zr_*x*_O_2_ (HZO) thin films.^8,13^ Recent studies, however, have unveiled a distinct family of rhombohedral (*r*) phases (space groups *R3*, *R3m*) that exhibit unique structural and electrical characteristics.^14^ These *r*-phases, though lying energetically above the monoclinic (*m*) ground state, offer enticing advantages: they show immediate wake-up-free ferroelectric switching (i.e., no initial cycling is required to activate polarization),^4,15^ significantly lower coercive fields,^4,16^ and improved compatibility with complex heterostructures (e.g., in tunnel junctions or multilayers).^17^ Such attributes directly address the aforementioned challenges, positioning the *r*-phase HZO as a promising avenue toward lower-voltage ferroelectric devices.

A landmark demonstration of the *r*-phase’s potential came from Wei et al.,^14^ who stabilized a polar *R3m* phase in epitaxial HZO under [111]-oriented compressive strain. The strained *R3m* films achieved a remanent polarization of ∼34 μC cm^–2^ without any wake-up process, underscoring the strong polar order of this phase. However, this strain-engineered ferroelectric showed limited endurance (on the order of 10^4^ switching cycles^18^), poorly below the >10^10^ cycles typical of *Pca2*_*1*_-based devices.^13^ Moreover, first-principles calculations indicated that a fully relaxed (strain-free) *R3m* structure may lose its polarization and develop soft-mode instabilities,^8^ highlighting the challenge of stabilizing this phase without external constraints. More recently, an intrinsic route to the *r*-phase was achieved via compositional tuning. Wang et al.^4^ demonstrated that Hf­(Zr)_1+*x*_O_2_ thin films—with a slight excess of cations intercalated into the lattice—can spontaneously form a stable polar *R3* phase. The intercalated Hf/Zr atoms expand the lattice and induce internal stress, which biases the crystal into the rhombohedral structure. Ferroelectric capacitors based on this *r*-phase Hf­(Zr)_1+*x*_O_2_ exhibited a low coercive field (∼0.65 MV cm^–1^), a remanent polarization of ∼22 μC cm^–2^, and endurance beyond 10^12^ cycles.^4^ This performance rivals state-of-the-art *o*-HZO devices and firmly reveals the *r*-phase as a strong contender for next-generation ferroelectric memory.

Despite this progress, a central question remains: can the *R3* phase—arising as a polar derivative of the parent fluorite lattice—exhibit intrinsic ferroelectricity without relying on intercalation? While cation intercalation has been shown to bias hafnia into the rhombohedral structure, such strategies introduce undesirable complexity, including lattice distortion, stress accumulation, and limited endurance.^4,16^ In this work, we address this challenge using density functional theory (DFT) and show that *R3*-HZO can emerge from intrinsic symmetry breaking of the fluorite framework and stabilize a robust polar ground state. This intrinsic route does not require intentional cation intercalation or external stress, and can coexist with previously established intercalation- and strain-based stabilization pathways. By mapping the energy landscape, switching barriers, and domain dynamics, we highlight its potential for low-voltage operation. We further evaluate extrinsic influences—such as multiphase coexistence and electrode-induced interfacial fields—on polarization stability and coercive field. Notably, the ferroelectric response in *R3*-HZO deviates from the conventional displacement-driven switching observed in *Pca2*_*1*_, revealing a distinct polarization mechanism rooted in its fluorite-derived topology. This work thus charts a symmetry-guided pathway toward energy-efficient ferroelectric memory based on the *R3* phase.

## 2 Results and Discussion

### 2.1 Intrinsic Polar Stabilization Metrics and Switching Pathways in the *r*-HZO Phase

Figure 1 summarizes the structural motifs and intrinsic polar response of a series of fluorite-derived rhombohedral (*R3*) polymorphs of HfO_2_, designed to isolate the microscopic mechanisms governing polarization stabilization and dielectric enhancement. To systematically probe the emergence of ferroelectricity under rhombohedral symmetry, we constructed three minimal 9-atom *R3* configurations (*R3*-I to *R3*-III) by introducing distinct symmetry-allowed Wyckoff displacements compatible with the *R3* space group. All structures share the same rhombohedral metric (*a* = *b* = c, α = β = γ = 88.71°) following Hu et al.,^8^ ensuring that differences in polar response arise solely from internal atomic rearrangements rather than lattice strain. After equation-of-state volume optimization, atomic coordinates were relaxed under fixed lattice parameters to preserve rhombohedral symmetry while allowing local inversion-symmetry breaking. Density-functional calculations were performed using Quantum ESPRESSO, with DFT+*U* corrections applied to the Hf and Zr *d* states to properly capture electronic localization effects.^19^ Computational details, symmetry validation, and convergence tests are provided in Supporting Information Sections S1–S3 and Figures S1–S3.

**Figure 1:**
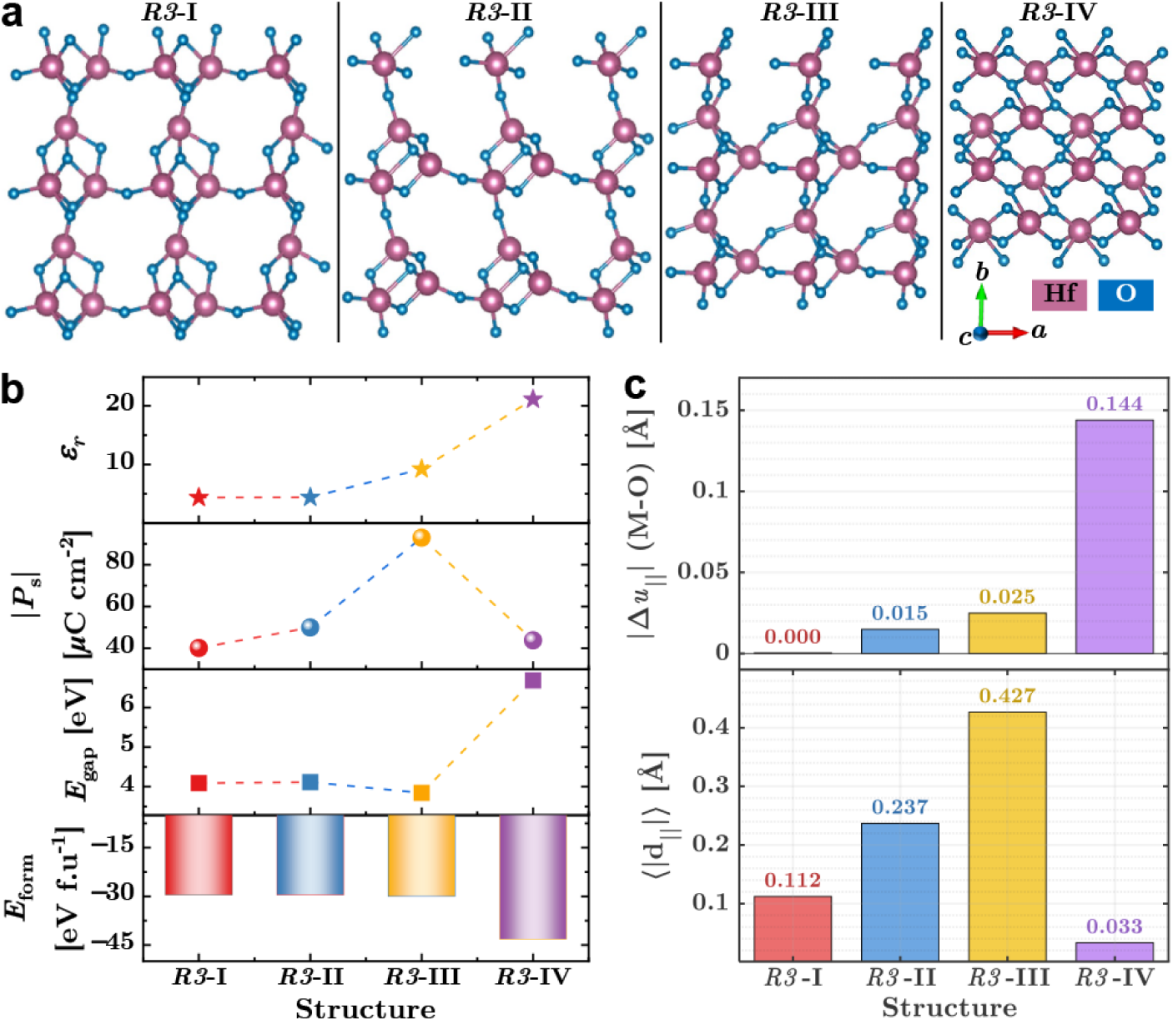
DFT+*U* comparison of polar *R3*-HfO_2_ configurations and coordinate-derived polarization metrics. (a) Atomic configurations shown in a 2 × 2 × 2 supercell. *R3*-I–*R3*-III contain 9 atoms per primitive unit cell, whereas *R3*-IV adopts a 12-atom cell. (b) Calculated *ε_r_*, Berry-phase spontaneous polarization |*P_s_* |, band gap *E*_gap_, and formation energy *E*_form_ for fully relaxed structures. (c) Coordinate-derived structural descriptors characterizing polar coherence and compensation: top, polar sublattice separation |Δ*u*_∥_| (cation–oxygen center-of-mass displacement projected along the pseudo-⟨111⟩ direction); bottom, average magnitude of local Hf-centered dipoles ⟨|*d*_∥_|⟩.

To quantitatively disentangle polarization enhancement from suppression across the *R3* polymorphs, we extracted two complementary coordinate-based descriptors from the fully relaxed structures. The first characterizes the amplitude of local polar distortions by defining Hf-centered dipoles,

1 $$d_{j}=r_{\mathrm{Hf},j}-\frac{1}{N_{j}}\underset{k\in \mathrm{nn}\left(j\right)}{\sum }r_{O,k}$$

where nn­(*j*) denotes the nearest-neighbor oxygen coordination shell of the *j*th cation. The average projected magnitude ⟨|*d*_∥_|⟩, obtained from *d*_*j*,∥_ = **d**_*j*_ × **ê**_111_, measures the strength of local polar distortions irrespective of their phase coherence. The second descriptor captures the net coherence of polar displacements through the relative shift between the cation and oxygen sublattices along the pseudo-⟨111⟩ direction,

2 $$\Delta u_{∥}=\left(r_{\mathrm{cat}}^{\mathrm{avg}}-r_{O}^{\mathrm{avg}}\right)\times {ê}_{111}$$

where $r_{\mathrm{cat}}^{\mathrm{avg}}$ and $r_{O}^{\mathrm{avg}}$ denote the mean positions of the Hf­(Zr) and oxygen sublattices, respectively.

As shown in Figure 1a, the three 9-atom *R3* polymorphs exhibit progressively enhanced Hf–O displacements along the pseudo-[111] axis from *R3*-I to *R3*-III. This evolution is reflected in Figure 1b by a monotonic increase in the Berry-phase spontaneous polarization *P*_*s*_ and a modest enhancement of the dielectric constant *ε*_*r*_. Notably, even the least distorted configuration (*R3*-I) sustains a sizable polarization of ∼40 μC cm^–2^, indicating that the fluorite-derived *R3* lattice exhibits an intrinsic instability toward a polar state. *R3*-II introduces cooperative octahedral tilting that reinforces polar alignment, while *R3*-III achieves the strongest coherent displacement, yielding the largest *P*_*s*_ among the 9-atom cells. Despite these sizable polarizations, all three compact *R3* variants exhibit relatively low dielectric constants (*ε*_*r*_ < 10), significantly smaller than those of strongly ferroelectric HfO_2_ phases (typically *ε*_*r*_ ∼ 20–40).^3,4^ This discrepancy indicates that while the minimal *R3* cells capture short-range polar distortions, they fail to accommodate the long-range cooperative oxygen rearrangements necessary to enhance lattice polarizability.

To capture such long-range effects, we constructed an extended *R3* structure with a 12-atom unit cell (*R3*-IV). As summarized in Figure 1c, *R3*-IV exhibits the largest polar sublattice separation Δ*u*_∥_, confirming that strong Hf–O off-centering persists in the extended network. However, unlike the monotonic increase observed from *R3*-I to *R3*-III, the spontaneous polarization *P*_*s*_ is significantly reduced in *R3*-IV relative to *R3*-III (Figure 1b), indicating that symmetry-compatible arrangements of locally polarized units substantially cancel the macroscopic dipole. This interpretation is further supported by the marked reduction in the net sublattice displacement *d*_∥_ (Figure 1c). However, at the same time, the extended configuration enables a large field-induced lattice response, resulting in a substantial enhancement of the dielectric constant in *R3*-IV. Therefore, the reduced polarization in *R3*-IV arises not from weakened local distortions, but from their spatial organization within the extended oxygen framework. Long-range oxygen rearrangements thus play a dual role: they renormalize the net polarization while simultaneously amplifying the dielectric response, revealing an intrinsic polarization–permittivity trade-off in fluorite-derived *R3* HfO_2_.

To further assess the energetic viability and compositional tunability of this behavior, we extended the analysis to the 12-atom *R3*-phase cells with varying Zr content. Figure 2a–e summarize the equation-of-state optimization and the resulting spontaneous polarization (*P*_*s*_), formation energy (Δ*E*_form_), dielectric permittivity (*ε*_*r*_), and band gap (*E*_gap_), alongside comparisons with the monoclinic (*P2*_1_ */c*) and orthorhombic (*Pca2*_1_) reference phases. Full crystallographic details are provided in Supporting Information Section S2.

**Figure 2:**
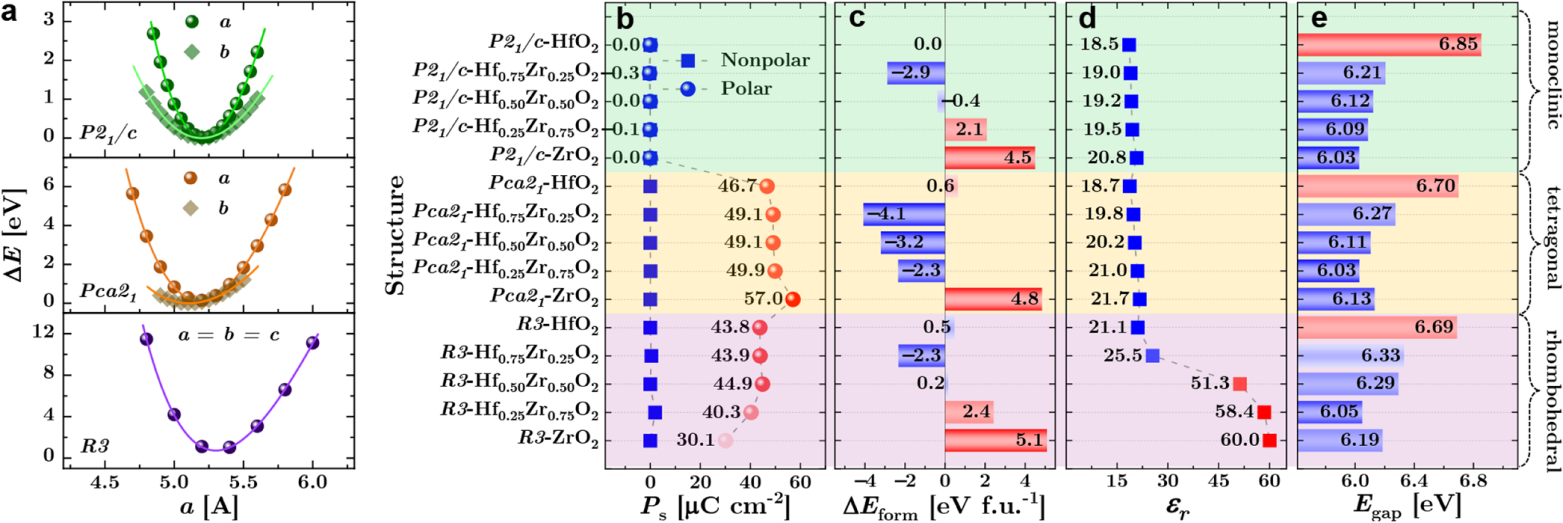
Symmetry-resolved equation-of-state and property evolution of ground-state fluorite-derived Hf_1–*x*_Zr*_x_* O_2_ polymorphs. (a) EOS fitting of lattice constants *a* and *b*. DFT+*U*-calculated (b) polarization *P_s_*, (c) formation-energy difference (Δ*E*_form_) relative to the *P2*_1_ */c*-HfO_2_ phase, (d) dielectric permittivity *ε_r_*, and (e) band gap *E*_gap_ of Hf_1–*x*_Zr*_x_* O_2_ with various symmetries.

As illustrated in Figure 2a, *R3*-HfO_2_ structures were generated by symmetrizing relaxed monoclinic configurations under the same rhombohedral metric. Consistent with prior studies,^8^ full relaxation often converges to nonpolar minima (Figure 2b), underscoring the inherent resistance of the fluorite-derived lattice to long-range polar order. However, when these nonpolar references are slightly perturbed (∼0.02 Å), the system spontaneously breaks inversion symmetry and relaxes into a polar *R3* configuration. The resulting *R3*-IV phase exhibits a favorable combination of moderate spontaneous polarization (*P*_*s*_ > 40 μC cm^–2^), a large dielectric constant (*ε*_*r*_ > 20), a wide band gap (*E*_gap_ ∼ 6 eV), and a substantially reduced formation energy relative to the compact 9-atom cells. Moreover, the dielectric response can be further enhanced by Zr substitution, collectively defining the key attributes of a technologically promising ferroelectric phase. The remaining open question is whether this polar state is energetically preferred over its nonpolar counterpart.

To quantify this dependence, we performed climbing-image nudged elastic band (NEB) calculations to resolve the polarization reversal pathways across various Zr concentrations (Figure 3a). For pure HfO_2_ (*x* = 0), the energy profile increases steeply, exhibiting a high barrier (>200 meV) toward the polar states and thus a clear preference for the nonpolar lattice. The introduction of Zr (*x* = 0.25) lowers the barrier but results in only metastable polarization along the transition path—indicating emergent yet frustrated symmetry breaking. At the critical composition (*x* = 0.5), a distinct lower potential barrier of ∼150 meV emerges, signifying the stabilization of a true ferroelectric state. In the Zr-rich limit (*x* = 1.0), the polar energy landscape deepens, indicating a more stable polar structure; however, the reversal toward the centrosymmetric reference state encounters a significantly larger switching barrier. The energy difference between the centrosymmetric and polar configurations (Δ*E*_centro–polar_), which identifies the composition-driven stabilization of the *r*-phase, is clearly reflected in Figure 3b. Hf-rich compositions (*x* = 0–0.25) exhibit large positive values (>198 meV), indicating that polar distortions are metastable. By contrast, Δ*E*_centro–polar_ becomes negative at *x* = 0.5 (−26.3 meV) and strongly favorable at *x* = 1.0 (−249.1 meV), confirming the emergence of a compositionally stabilized polar ground state.

**Figure 3:**
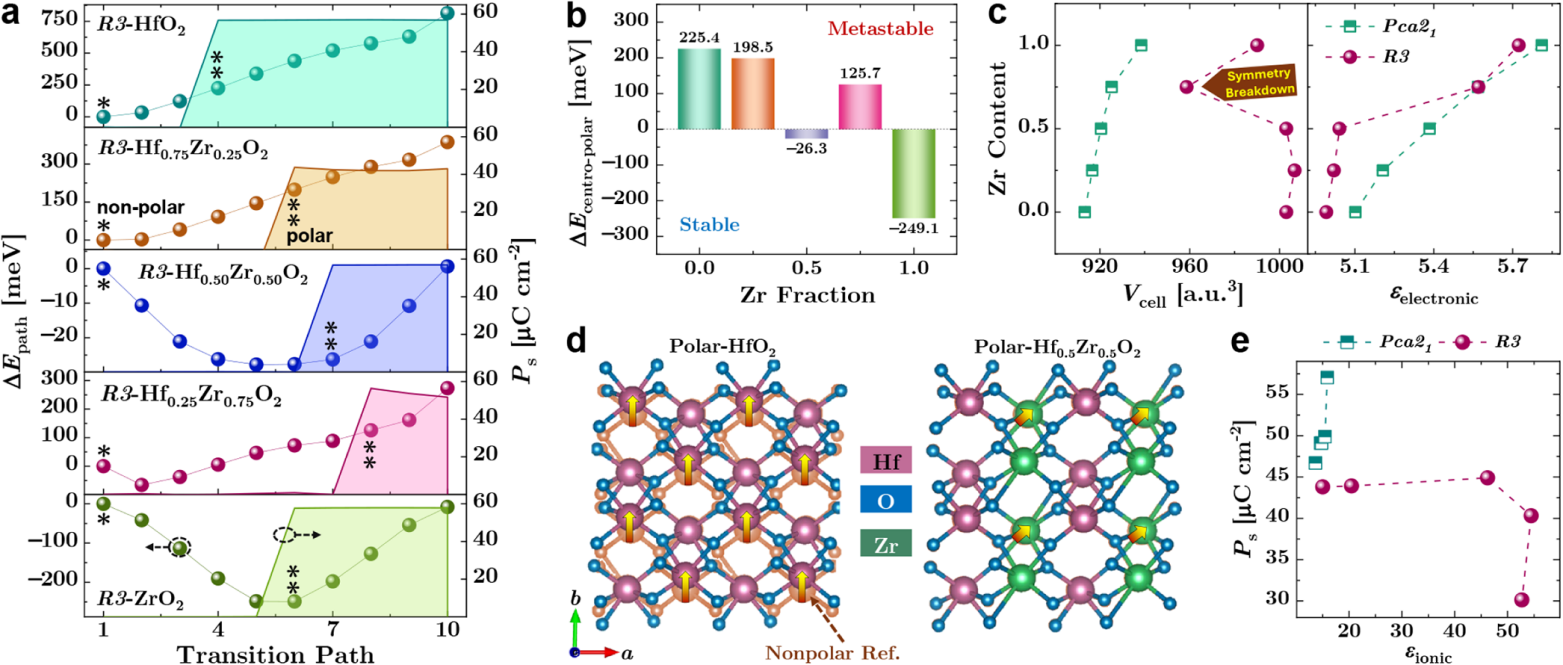
Energy landscape and polarization switching in*R3*-Hf_1–*x*_Zr*_x_* O_2_polymorphs. (a) NEB energy profiles (left axis) and corresponding polarization evolution (right axis) for *r*-HZO, illustrating the nonpolar–to–polar switching pathway. (b) Compositional dependence of the *r*-phase energy landscape as a function of Zr content. (c) Evolution of unit-cell volume (*V*_cell_) and electronic permittivity (*ε*_electronic_) with Zr substitution, comparing the *r*- and *o*-phases. (d) Atomistic representations of the nonpolar–to–polar switching pathway for representative compositions: *r*-HfO_2_ and *r*-Hf_0.5_Zr_0.5_O_2_. (e) Polarization plotted against ionic permittivity (*ε*_ionic_), contrasting the *o*- and *r*-phases characteristics.

The structural and electronic descriptors presented in Figure 3c reveal the microscopic origin of the composition-driven crossover in phase stability. For the *o*-phase (*Pca2*_*1*_), both the unit-cell volume (*V*_cell_) and the electronic permittivity (*ε*_electronic_) evolve nearly monotonically with increasing Zr content, reflecting a conventional Vegard-like response to alloying.^20^ In sharp contrast, the *R3*-phase exhibits a pronounced nonmonotonic behavior, with abrupt deviations near *x* ≈ 0.75. This anomaly signals a breakdown of simple volumetric or electronic trends and instead points to an intrinsic symmetry-renormalization process unique to the rhombohedral lattice. The absence of smooth scaling in *R3* rules out volumetric expansion or purely electronic screening as the stabilization mechanism, underscoring the central role of local symmetry-breaking displacements in enabling ferroelectricity.

Figure 3d provides atomistic insight into the microscopic origin of metastable switching in HfO_2_ and its stabilization at *x* = 0.5. In pure *R3*-HfO_2_, antipolar Hf–O displacements effectively cancel the macroscopic polarization, yielding a quasi-nonpolar ground state despite the presence of local dipoles. Consequently, inducing a polar transition requires substantial energy, leading to a large switching barrier. By contrast, at *x* = 0.5, Zr incorporation promotes coherent cation shifts along the ⟨111⟩ axis, aligning local dipoles and producing a robust macroscopic polarization. This compositional tuning markedly reduces the switching barrier and stabilizes an energetically favorable ferroelectric ground state—illustrating how moderate Zr substitution facilitates polar distortion and enhances switchability in *r*-HZO.

This compositional dependence is further illustrated in Figure 3e, which plots the *P*_*s*_ as a function of the ionic dielectric constant (*ε*_ionic_). Despite its relatively low overall dielectric constant, the *o*-phase (*Pca2*_*1*_) maintains a consistently high *P*_*s*_ that increases nearly monotonically with *ε*_ionic_—a hallmark of a conventional displacement-type ferroelectric response following a Vegard-like compositional trend.^20^ In contrast, the *r*-phase (*R3*) exhibits a substantially higher ionic dielectric constant and a distinct nonmonotonic behavior, wherein *P*_*s*_ initially increases with *ε*_ionic_ before sharply decreasing at higher values. This anomaly signals a crossover from a displacement-driven to a symmetry-governed polarization mechanism, underscoring the unconventional polar coupling intrinsic to the *R3* lattice.

Therefore, these results uncover a distinct, compositionally activated route to ferroelectricity in *r*-HZO. Unlike the conventional tetragonal-to-orthorhombic field-induced transition in hafnia-based ferroelectrics, polarization in the *R3* phase emerges intrinsically through spontaneous symmetry breaking within the same lattice group. Nonetheless, as shown in Figure S4, this polar state is also continuously accessible via symmetry-lowering distortions from the tetragonal *P*4_2_/*nmc* configuration. Zr substitution thus serves as a tunable order parameter that lowers the energy barrier and stabilizes the polar ground state without the need for intercalants, interfacial fields, or external strain.

### 2.2 HRTEM and FFT-Based Experimental Identification of the *r*-Phase

To experimentally verify the structural emergence of the rhombohedral *R3* phase in Hf_0.5_Zr_0.5_O_2_, HZO capacitor stacks were fabricated by atomic-layer deposition followed by postdeposition annealing (see Supporting Information, Section S5 and Figure S5). High-resolution transmission electron microscopy (HRTEM) resolves lattice-fringe contrast arising from coherent electron scattering, enabling crystallographic analysis at the nanometer scale. Fast Fourier transforms (FFTs) of the HRTEM images convert real-space lattice periodicities into reciprocal-space information, where peak positions directly yield interplanar spacings (*d*-spacings). To enhance sensitivity to weak and orientation-dependent reflections and to suppress isotropic background scattering, we employed a direction-selective (sector-masked) FFT approach. As illustrated in Figure 4a,c, FFT intensity was integrated within a narrow angular sector (−104.7° ± 4.0°), followed by sector-integrated line profiles extracted along two symmetry-related angular slices at 138° ± 10° and 244° ± 10° (Figure 4b,d). This procedure stabilizes peak positions and significantly improves discrimination among closely related HZO polymorphs.^21–24^

**Figure 4:**
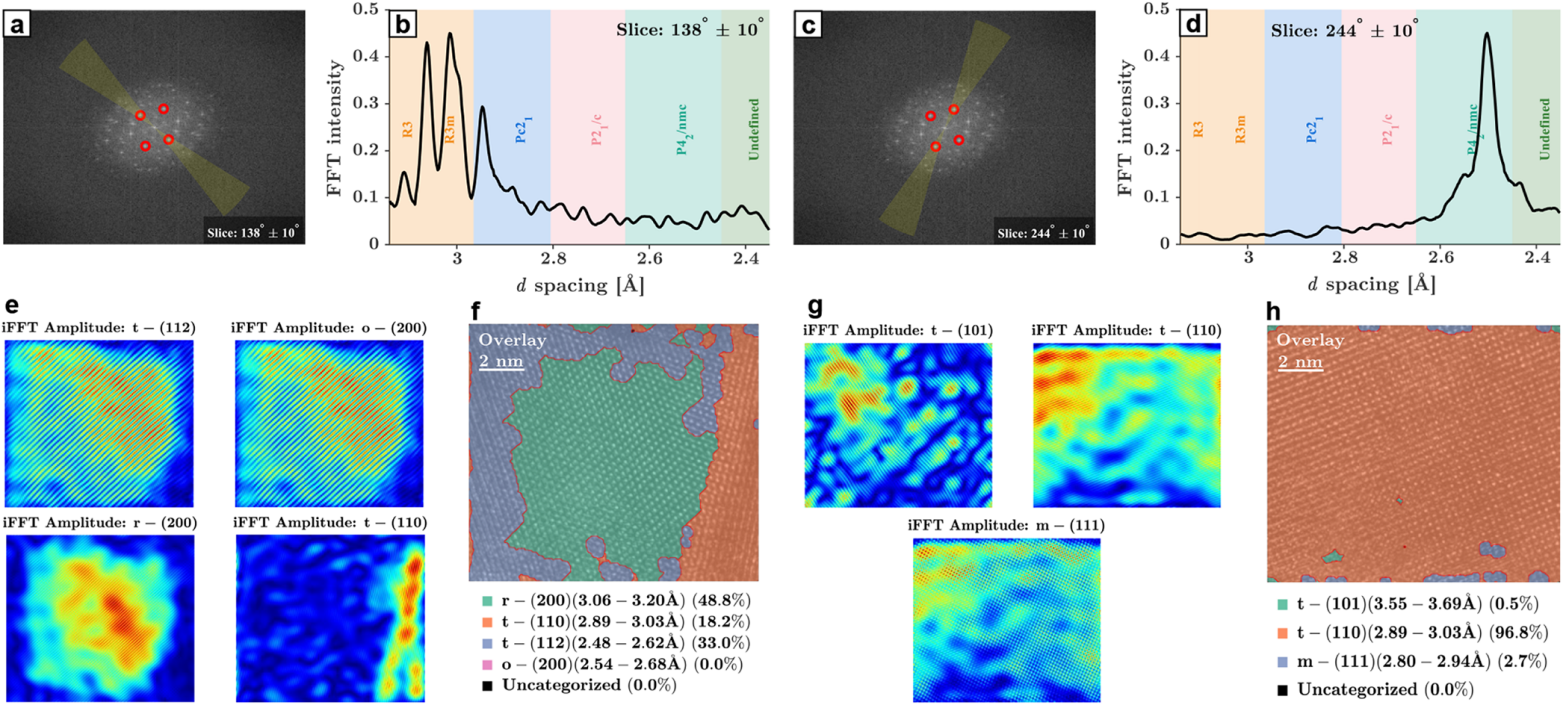
Sector-masked FFT and phase-selective iFFT polymorph mapping in HZO. (a,c) FFTs from the indicated regions of interest (ROIs) with a sector mask (−104.7° ± 4.0°); selected reflections for iFFT are marked (red). Intensity is integrated over angular slices at 138° ± 10° (b) and 244° ± 10° (d). (b,d) Sector-integrated FFT intensity vs *d*-spacing with diagnostic windows for *r* (*R3*), *o* (*P*2_1_/*c*), *t* (*P*4_2_/*nmc*), and undefined ranges. (e,g) Phase-selective iFFT amplitude maps. (f,h) Dominance overlays with area fractions (left: *r*-dominant ROI; right: *t*-dominant ROI).

Using reference structures obtained from EOS-based DFT optimization together with established crystallographic databases, this approach enables quantitative discrimination between closely related rhombohedral polymorphs, particularly *R3* and *R3m*. Table 1 summarizes the DFT-predicted *d*-spacings for the *r*-phase *R3* and the *o*-phase *Pca2*_1_, along with the *R3m* phase reported in the literature.^4^ These values serve as crystallographic benchmarks for identifying the experimentally observed polar phase. The (111)-family *d*-spacings are 3.13 Å for *R3* and 3.07 Å for *R3m* (indicated by dashed lines in Figure 4), whereas the *Pca2*_1_ phase exhibits a distinctly smaller value of ∼2.97 Å, corresponding to the blue-shaded region. Other competing polymorphs occupy well-separated *d*-spacing ranges: 2.75–2.85 Å for the monoclinic *P2*_1_ */c* (*m*) phase (light-red shaded) and 2.55–2.65 Å for the tetragonal *P4*_2_ */nmc* (*t*) phase (green shaded).^23,24^

**Table 1:** DFT-Calculated Bulk *d*-Spacings (Å) of Key Low-Index Planes for Polar Hf_0.5_Zr_0.5_O_2_ Polymorphs

			*R3m* ^4^	
hkl	*Pca2*_1_	*R3*	Hf/Zr-rich	Stoichiometric
(100)	5.22	5.30	–	–
(110)	3.65	3.79	–	–
(111)	2.97	3.13	3.07	2.91
(200)	2.61	2.65	–	–
(220)	1.83	1.89	–	–

The sector-integrated FFT profiles exhibit a pronounced intensity maximum at *d* ≈ 3.13 Å, corresponding to the *r*-(111) reflection of the *R3* phase (Figure 4b). This peak is well separated from the dominant orthorhombic and tetragonal reflections near *d* ≈ 2.96–2.97 Å [*o*-(111), *t*-(110)] and from higher-*d* orthorhombic reflections near 3.65 Å [*o*-(110)], providing a robust reciprocal-space anchor for distinguishing *R3* from distorted *Pca*2_1_-like phases. By contrast, the complementary angular slice at 244° ± 10° (Figure 4d) is dominated by intensity within the tetragonal window, demonstrating that the film is locally multiphase and underscoring the necessity of directional and multipeak criteria rather than single-spot phase assignment.

Table 2 summarizes the DFT-based structural fingerprints used for experimental phase identification, comparing characteristic *R3* reflections with the nearest competing reflections from *o*-, *t*-, and *m*-polymorphs. The quantified |Δ*d*| values explicitly distinguish robust anchor reflections (e.g., *r*-(111)) from marginal cases that require multipeak confirmation and real-space validation.

**Table 2:** DFT-Based Structural Fingerprints Used for Experimental Identification of the *R3* phase^a^

*R3* plane (DFT)	*d*_DFT_ (Å)	Nearest competing plane	*d*_DFT_ (Å)	|Δ*d*| (Å)
*r*-(110)	3.79	*o*-(110)	3.65	0.14
*r*-(111)	3.13	*o*-(111)/*t*-(110)	2.97/2.96	0.16–0.17
*r*-(200)	2.65	*o*-(200)/*m*-(200)	2.61/2.59	0.04–0.06
*r*-(220)	1.89	*m*-(020)/*o*-(202)	1.84/1.83	0.05–0.06

a DFT-Calculated *d*-spacings of characteristic *R3* reflections are compared with the nearest competing reflections from orthorhombic (*o*), tetragonal (*t*), and monoclinic (*m*) polymorphs. The corresponding |Δ*d*| values define robust anchor reflections and marginal cases requiring multi-peak confirmation.

To validate that the reciprocal-space assignments correspond to physically meaningful real-space domains, phase-selective inverse FFT (iFFT) reconstructions were performed. As shown in Figure 4e,g, reflections assigned to individual *r*-, *t*-, *o*-, and *m*-phases reconstruct spatially coherent lattice-fringe patterns. The dominance overlays in Figure 4f,h further quantify the local phase fractions within each region of interest. In the *r*-dominant region (Figure 4f), nearly half of the area is assigned to the *R3*-related reflection window, whereas the comparison region (Figure 4h) is overwhelmingly *t*-phase dominant. Random noise or marginal peak overlap does not produce contiguous domains in the iFFT maps, providing essential real-space cross-validation of the FFT-based phase assignment.

Overall, the combination of direction-selective FFT integration, quantitative *d*-spacing matching, and phase-selective iFFT domain coherence establishes a robust, multiconstraint identification of the *r*-phase. This approach explicitly mitigates the risk that local strain or small lattice distortions mimic rhombohedral symmetry and confirms the presence of intrinsic, intercalation-free *R3* ordering in the HZO films.

### 2.3 Thickness- and Interface-Driven Stabilization of the *r*-HZO Phase

Beyond intrinsic lattice energetics, the realization of the *r*-HZO phase in thin films is strongly governed by extrinsic phase-selection conditions, most notably nanoscale thickness confinement and interfacial boundary conditions. These factors do not generate polarization within the *r*-phase itself; rather, they bias the relative stability of competing polymorphs during film formation by controlling local strain accommodation, interphase-boundary energetics, and electrostatic continuity at electrode interfaces. Their combined influence therefore determines the fraction and spatial distribution of the *r*-phase that emerges in real devices, as well as the effective coercive field measured at the capacitor level.

Phase-selective inverse FFT (iFFT) mapping, combined with geometric phase analysis (GPA), was employed to translate the sector-averaged FFT phase identification into real-space domain distributions. Representative cross-sectional HRTEM–GPA results for SiO_2_/HZO/W stacks are shown in Figure 5a, where coexisting *r*, *t*, and minor *m* domains are spatially resolved. As the HZO thickness is reduced from 15 to 7.5 nm, the areal fraction of *r*-phase domains increases markedly (Figure 5b), accompanied by a suppression of the orthorhombic *o*-phase. This experimentally observed trend is qualitatively reproduced by DFT calculations (Figure 5c), which show a monotonic lowering of the relative total energy of the *R3* phase with decreasing thickness, indicating a thermodynamic driving force for *r*-phase stabilization under nanoscale confinement.

**Figure 5:**
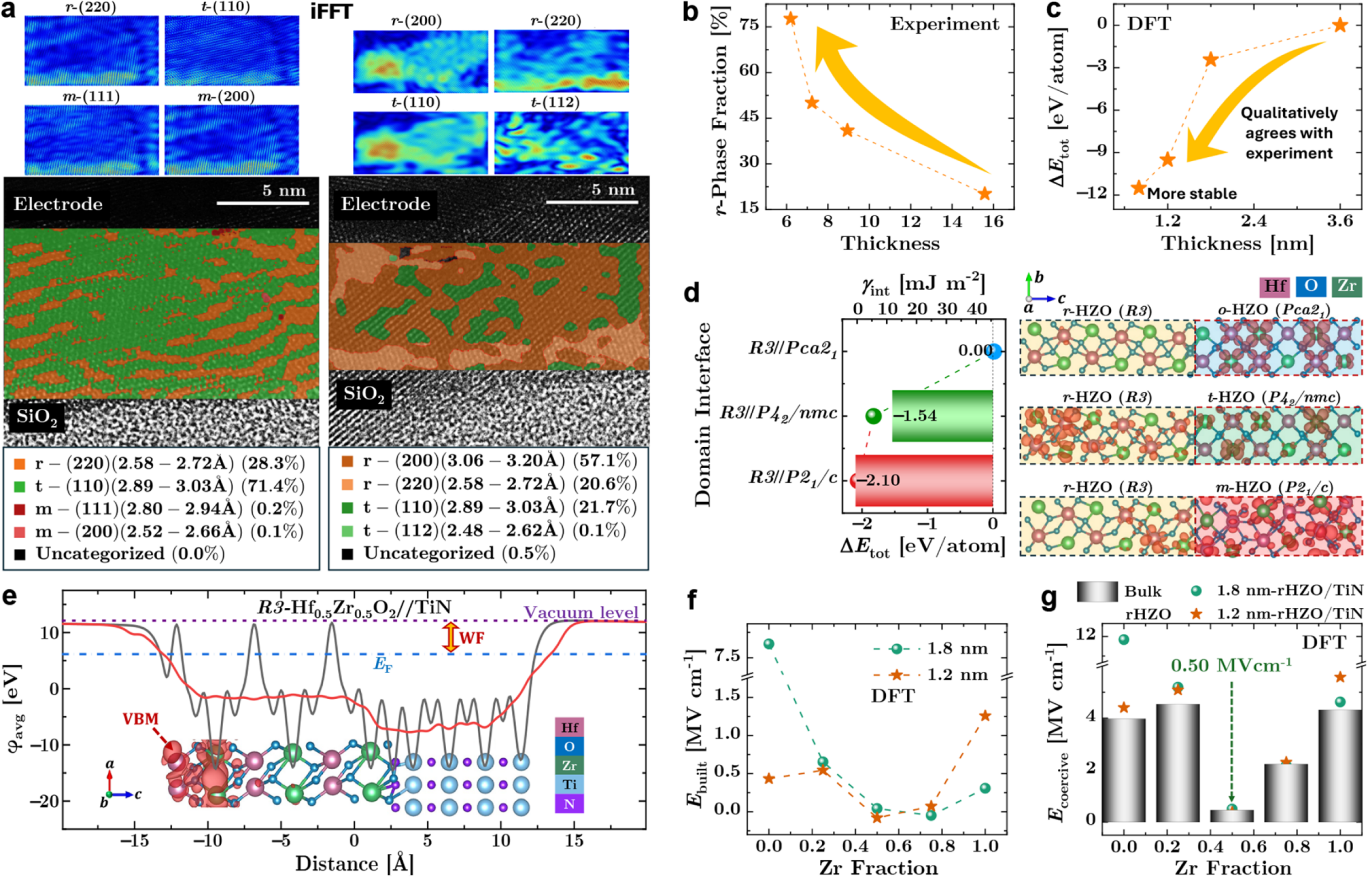
Thickness- and interface-dependent stabilization of rhombohedral*r*-HZO. (a) Phase-selective iFFT maps and dominance overlays showing coexisting *r*/*t*/*m* domains. (b) Experimental *r*-phase fraction as a function of film thickness. (c) DFT-calculated thickness-dependent Δ*E*_tot_, qualitatively consistent with b. (d) Interfacial energetics (γ_int_ and Δ*E*_tot_) and corresponding VBM isosurfaces for *R3* interfaces with competing polymorphs. (e) Planar-averaged electrostatic potential across the *R3*-HZO/TiN interface (inset: VBM isosurfaces). (f) Built-in electric field *E*_built_ versus Zr fraction for ultrathin HZO/TiN. (g) Comparison of intrinsic and electrode-modified coercive fields *E*_coercive_ as a function of Zr fraction.

At the microstructural level, the energetic compatibility of interphase boundaries further governs local phase coexistence. Figure 5d compares the interfacial energies (γ_int_) and relative total energies for interfaces between *R3* and competing polymorphs. Interfaces between *r*-HZO and either the tetragonal (*P4*_2_ */nmc*) or monoclinic (*P2*_1_ */c*) phases exhibit comparatively low interfacial energies and smooth valence-band-maximum (VBM) profiles, indicating good structural and electrostatic compatibility. In contrast, interfaces between *R3* and the orthorhombic *Pca2*_1_ phase display substantially higher interfacial energies and abrupt VBM discontinuities, reflecting pronounced dipole mismatch. These energetics explain why *r*-domains preferentially coexist with *t*- and *m*-regions, while the *o*-phase is progressively suppressed in thinner films.

Electrode selection further modulates *r*-phase stability by altering interfacial electrostatics and screening conditions. Figure 5e shows the planar-averaged electrostatic potential across an *R3*-HZO/TiN interface, revealing a smooth potential profile and a reduced effective work-function mismatch. The resulting built-in electric field, extracted from the potential gradient, is summarized in Figure 5f as a function of Zr fraction. Near equiatomic composition, the built-in field is strongly suppressed, consistent with enhanced electrostatic screening of polar distortions at the TiN interface. This electrostatic environment directly impacts switching behavior: Figure 5g shows that the intrinsic low coercive field predicted for *R3*-HZO (∼0.5 MV cm^–1^) is preserved or further reduced in TiN-contacted devices, whereas less effective screening leads to higher apparent coercive fields.

Hence, these results demonstrate that the stabilization of the *r*-HZO phase arises from a cooperative interplay between thickness-driven confinement, favorable interphase-boundary energetics, and electrode-induced electrostatic screening. This synergy explains the experimentally observed enhancement of *r*-phase fraction, the suppression of competing *o*-domains, and the realization of low-coercive-field ferroelectricity in ultrathin HZO devices.

### 2.4 Optimal Regime of Low Coercive Field in the *r*-HZO Phase

Having identified the structural and interfacial origins of *r*-phase stabilization, we now turn to how its exceptionally low coercive field emerges from the cooperative balance between intrinsic lattice energetics and interfacial electrostatics. To establish a consistent baseline, we define the energy difference between centrosymmetric and polar distortions within the same *R3* symmetry as

3 $$\Delta E_{\mathrm{centro}-\mathrm{polar}}≡E_{\mathrm{polar}}-E_{\mathrm{centro}}$$

where *E*_polar_ and *E*_centro_ are the total energies of the relaxed polar and centrosymmetric structures, respectively. A negative Δ*E*_centro–polar_ indicates spontaneous symmetry breaking toward a polar ground state without external drivers such as intercalants or interfacial strain. As shown in Figure 3, Δ*E*_centro–polar_ becomes negative at *x* = 0.5 and *x* = 1.0, identifying these compositions as intrinsically stable ferroelectric candidates. When metallic electrodes are introduced, the thermodynamic landscape is further renormalized by electrostatic coupling across the interface. In Landau–Ginzburg–Devonshire theory, this contribution enters the free energy as −∫ *P*(*z*) *E*_built_(*z*) d*z*,^25,26^ which in our formulation modifies the intrinsic double-well depth as

4 $$\Delta E_{\mathrm{eff}}\approx \Delta E_{\mathrm{centro}-\mathrm{polar}}-\int_{0}^{d}P(z)\;E_{\mathrm{built}}(z)\;dz$$

where *d* is the film thickness, *P*(*z*) is the polarization profile, and *E*_built_ is the built-in field. Physically, the built-in field tilts the double-well potential, reducing the barrier height for switching along the field-assisted direction and increasing it for the opposite (field-opposed) direction. This asymmetry gives rise to imprinted hysteresis loops and manifests as shifted coercive fields:^25,26^

5 $$E_{\mathrm{coercive}}^{\mathrm{eff}}=E_{\mathrm{coercive}}^{\mathrm{intrinsic}}\pm E_{\mathrm{built}}$$

with the minus sign corresponding to switching aided by the built-in field and the plus sign to switching against it.

As illustrated in Figure 5e–g, the electrostatic boundary conditions imposed by the electrode can substantially modify the effective switching field, even when the intrinsic lattice energetics remain unchanged. For ultrathin HZO/TiN stacks, the planar-averaged electrostatic potential (Figure 5e) reveals that a potential drop of ∼1.4 eV across a 1.8 nm film can generate a sizable built-in field. This field, quantified in Figure 5f, reaches several MV cm^–1^ for off-stoichiometric compositions and acts opposite to the spontaneous polarization, thereby increasing the effective switching barrier. As a consequence, although the intrinsic U-shaped dependence of *E*_coercive_ on Zr content predicted by DFT is preserved, most compositions exhibit an elevated effective coercive field $E_{\mathrm{coercive}}^{\mathrm{eff}}$ due to enhanced depolarization fields under realistic electrode boundary conditions (Figure 5g). In contrast, the near-equimolar composition (*x* = 0.5) remains largely immune to this interfacial penalty, consistently yielding the lowest $E_{\mathrm{coercive}}^{\mathrm{eff}}$ . This robustness is directly linked to the minimized built-in field extracted at *x* = 0.5 (Figure 5f), which originates from smooth band alignment and reduced charge discontinuity at the *R3*-HZO/TiN interface, as evident from the electrostatic potential and charge-distribution profiles in Figure 5e (inset). Microscopically, the more delocalized valence-band charge density promotes continuous interfacial bonding and efficient electrostatic screening, thereby suppressing depolarization fields and enabling reversible polarization switching at minimal energetic cost. As a result, the effective coercive field is minimized to ∼0.50 MV cm^–1^ for *x* = 0.5, defining an optimal composition for low-voltage ferroelectric operation that remains stable under realistic electrode contact.

Altogether, these results converge into a coherent picture in which intrinsic lattice energetics set the baseline ferroelectric stability, while interfacial electrostatics modulate the effective coercive field. At the 50:50 Hf:Zr ratio, these effects act synergistically—negative Δ*E*_centro–polar_, minimized built-in fields, smooth band alignment, and efficient electrode screening—to produce a stable *r*-phase ferroelectric with an exceptionally low coercive field.

### 2.5 Electrical Signatures of Intrinsic Ferroelectric Switching in the *r*-Phase

The *P*–*E* hysteresis loops measured before and after wake-up cycling (Figure 6a) show that the pristine *r*-phase–dominant device already exhibits a clear ferroelectric response with a low coercive field, while electrical conditioning further improves loop symmetry and remanent polarization. Consistently, the corresponding *C*–*E* characteristics (Figure 6b) evolve toward a more pronounced butterfly shape after wake-up, indicating enhanced dielectric susceptibility near the coercive field.

**Figure 6:**
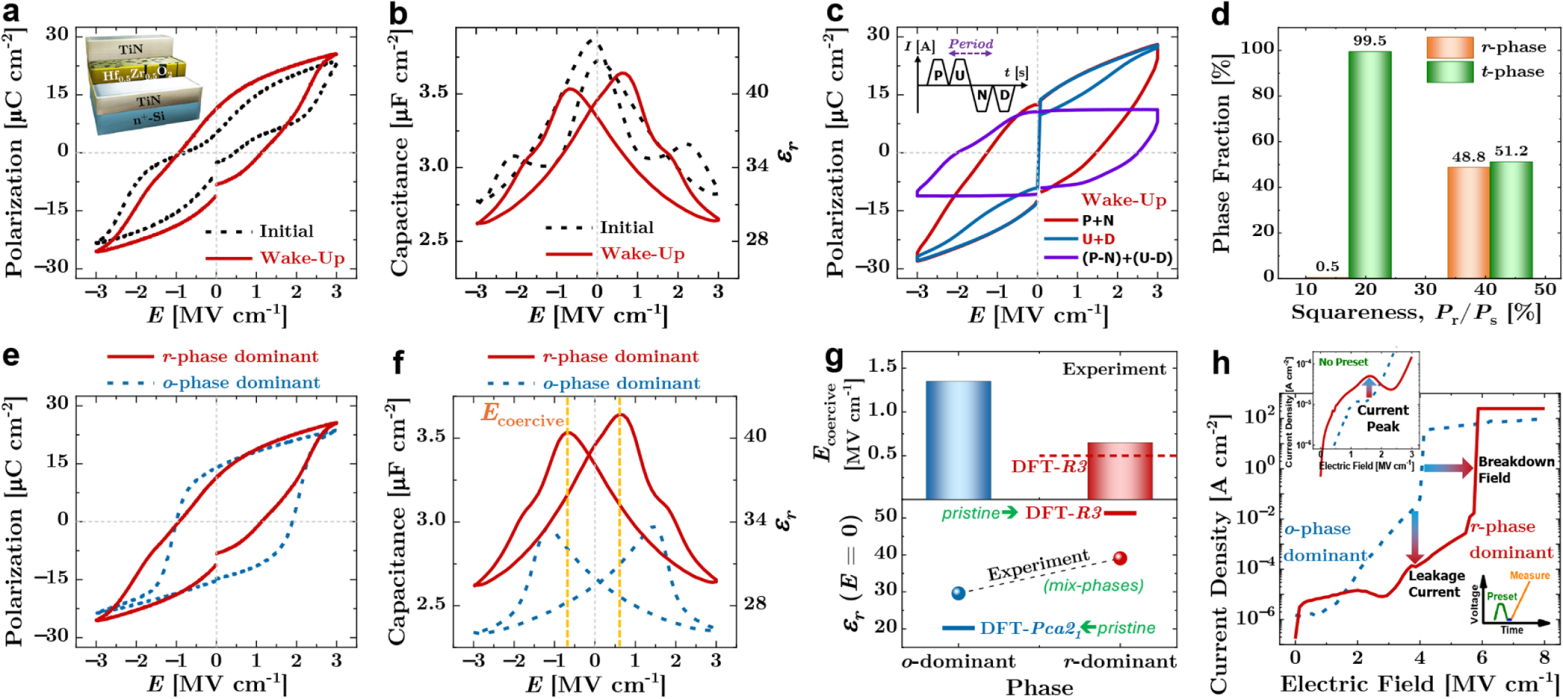
Electrical verification of intrinsically low-coercive-field switching in*r*-HZO. (a,b) *P*–*E* and *C*–*E* loops of an *r*-dominant capacitor before/after wake-up (inset: TiN/HZO/TiN/*n* ^+^-Si), showing improved symmetry and a more pronounced butterfly response after conditioning. (c) PUND separation of switching (P+N), nonswitching (U+D), and remanent [(P–U) + (N–D)] components, confirming intrinsic ferroelectric switching. (d) Loop squareness (*P_r_*/*P_s_*) versus phase fraction from phase-resolved iFFT, linking nonideal squareness to residual phase mixing. (e,f) *r*-dominant (red) versus *o*-dominant (blue) devices, showing lower *E*_coercive_ and higher permittivity for the rhombohedral phase. (g) Experiment–DFT comparison of *E*_coercive_ and *ε_r_*, showing qualitative agreement with the low-barrier *R3* state. (h) Leakage current density vs field, showing reduced leakage, a switching-related current peak, and higher breakdown in the *r*-dominant device.

To rigorously distinguish ferroelectric switching currents from leakage contributions, pulsed polarization measurements were performed using the standard PUND protocol (Figure 6c). The corresponding PUND response prior to wake-up conditioning is provided in Figure S6. After wake-up cycling, the extracted remanent polarization component, [(P–U) + (N–D)], exhibits a robust and fully reversible switching signal, unambiguously confirming the intrinsic ferroelectric origin of the measured hysteresis loops.

Figure 6d further correlates the electrical loop squareness (*P*_*r*_/*P*_*s*_) with the local phase composition obtained from phase-resolved iFFT analysis. The *r*-phase–dominant device exhibits a substantially higher squareness (∼49%) compared with the *t*-phase–dominant reference (<1%), establishing a direct link between macroscopic electrical behavior and the underlying crystallographic phase fraction. This relationship indicates that deviations from ideal loop squareness arise primarily from residual phase mixing associated with the polycrystalline nature of the film, rather than from intrinsic limitations of *r*-phase ferroelectricity. This behavior contrasts with epitaxially strained *R3m* films, which have been reported to achieve remanent polarizations of ∼34 μC cm^–2^ without a wake-up process.^14^ Importantly, the *R3* phase studied here emerges through an intrinsic route, without externally imposed strain or intentional intercalation.

The wake-up behavior in the *r*-phase–dominant device is attributed to two concurrently operating mechanisms. First, redistribution of charged defects (e.g., oxygen vacancies) depins preexisting ferroelectric domains and improves switching symmetry, in line with the well-established wake-up behavior of HfO_2_-based ferroelectrics.^1,3^ Second, a partial field-induced crystallographic transformation from the nonpolar tetragonal (*P*4_2_/*nmc*) phase to the polar rhombohedral (*R*3) phase increases the effective switchable fraction, as evidenced by the decrease in *t*-phase fraction and the corresponding increase in *r*-phase fraction after cycling (Figure 6d). This phase-transformation pathway is further supported by the NEB-calculated energy landscape (Figure S4), in which the *P*4_2_/*nmc* configuration occupies a saddle-like position accessible under cyclic electric fields.

### 2.6 Experimental Verification of Low-Coercive-Field Ferroelectricity, Enhanced Permittivity, and Electrical Robustness in the *r*-Phase

Figure 6e–h summarize the key experimental signatures differentiating the electrical responses of *r*-phase–dominant (red solid curves) and *o*-phase–dominant (blue dashed curves) HZO capacitors, with direct comparison to DFT predictions provided in Figure 6g. The polarization–electric-field (*P*–*E*) hysteresis loops in Figure 6e show that the *r*-phase–dominant device exhibits a well-developed ferroelectric loop with a markedly lower coercive field compared with the *o*-phase–dominant counterpart. Although the loop is not perfectly square, its nearly symmetric opening and stable remanent polarization indicate robust and reversible switching, consistent with the low energy barrier predicted for the *R3* phase. The capacitance–electric-field (*C*–*E*) characteristics in Figure 6f further support this behavior: the *r*-phase–dominant capacitor exhibits a butterfly like hysteretic curve with a significantly higher dielectric constant than the *o*-phase–dominant one.

Figure 6g quantitatively correlates these measurements with DFT results. First-principles calculations predict a low coercive field of ∼0.50 MV cm^–1^ and a large dielectric constant of *ε*_*r*_ = 51.3 for pristine Hf_0.5_Zr_0.5_O_2_ in the rhombohedral *R3* phase (red lines). Experimentally, the *r*-dominant HZO film closely follows these theoretical trends, exhibiting a low coercive field (*E*_coercive_ = 0.65 MV cm^–1^) and an enhanced dielectric constant (*ε*_*r*_ ≈ 39.1 at zero field), consistent with the predicted polarization-switching softness and strong ionic contribution of the *R3* lattice. In contrast, the *o*-phase–dominant device shows a higher coercive field (*E*_coercive_ = 1.35 MV cm^–1^) and a smaller dielectric constant (*ε*_*r*_ ≈ 29.6), consistent with the intrinsically larger switching barrier and weaker dielectric response of the *Pca2*_*1*_ phase (*ε*_*r*_ = 20.2 from DFT). The slight deviation from ideal theoretical values is attributed to residual mixed *r* + *o* + *t* + *m* microdomains revealed by GPA analysis (Figure 5).

Finally, leakage-current and breakdown measurements (Figure 6h) demonstrate that the *r*-phase–dominant devices simultaneously exhibit reduced leakage current, a clear switching-related current peak near the coercive field, and a higher breakdown field. Importantly, this improved electrical robustness is maintained over a wide range of voltage sweep rates, as confirmed by the sweep-rate–dependent I–V characteristics (Figure S7) and triangular-wave *P*–*V* measurements (Figure S8). The consistency of these responses across different sweep conditions indicates that the observed low leakage and stable switching behavior are intrinsic to the *r* phase rather than measurement-rate artifacts. These characteristics closely mirror prior reports on rhombohedral hafnia-based ferroelectrics^4^ and further confirm that the *r* phase combines intrinsically soft polarization switching with superior electrical robustness.

## 3 Experimental Section

Metal–ferroelectric–metal (MFM) capacitors were fabricated on 6-in. *n* ^+^-Si substrates using a 30 nm TiN bottom electrode deposited by physical vapor deposition (PVD), a 10 nm Hf_0.5_Zr_0.5_O_2_ (HZO) layer grown by atomic layer deposition (ALD), and a 50 nm TiN top electrode deposited by PVD and patterned lithographically; crystallization was then achieved by rapid thermal annealing (RTA) at 500°C for 60 s in N_2_, with further fabrication details provided in Supporting Information Section S5. To correlate device structure with functional phase response, high-resolution transmission electron microscopy (HRTEM) was performed on cross-sectional specimens, where direction-selective fast Fourier transforms (FFTs) with sector masks were used to extract *d*-spacing profiles, phase-selective inverse FFTs (iFFTs) were employed to reconstruct real-space domain maps, and geometric phase analysis (GPA) was applied to obtain local strain and phase-fraction information; reference *d*-spacings were derived from EOS-optimized DFT structures. Electrical characterization was then carried out at room temperature using polarization–electric-field (*P*–*E*) hysteresis and capacitance–voltage (*C*–*V*) measurements with triangular waveforms at 1–10 kHz, pulsed polarization (PUND) measurements with 1–10 μs pulse widths to separate switching and nonswitching contributions, and DC I–V sweeps with sweep times of 0.1–100 ms, while the maximum applied fields were limited to ±3–4 MV cm^–1^ and electrical wake-up cycling was applied prior to postconditioning measurements.

## 4 Conclusion

This study resolves the central question of whether the rhombohedral (*r*) phase of hafnia can sustain intrinsic ferroelectricity without intercalation or externally imposed strain. First-principles calculations demonstrate that fluorite-derived Hf_0.5_Zr_0.5_O_2_ with *R3* symmetry stabilizes a robust polar ground state, characterized by a large spontaneous polarization (*P*_*s*_ = 44.9 μC cm^–2^), high dielectric permittivity (*ε*_*r*_ = 51.3), an ultralow switching barrier (27.8 meV), and a remarkably small coercive field (0.46 MV cm^–1^ intrinsically and ∼0.50 MV cm^–1^ under electrode boundary conditions). Importantly, these properties emerge without intentional intercalants or external stress, revealing that *R3* can serve as an intrinsically ferroelectric phase within the fluorite-derived hafnia framework, representing an additional route alongside previously reported intercalation-stabilized rhombohedral structures formed under different growth conditions. Experimental observations directly corroborate these predictions. GPA-based phase analysis reveals substantial *r*-phase fractions exceeding 50% in ultrathin films, demonstrating that the rhombohedral polymorph can become dominant within a mixed *r* + *o* + *t* + *m* microstructural environment. Interfacial-energy analysis further shows that *r* domains are preferentially stabilized when adjacent to tetragonal and monoclinic regions rather than orthorhombic neighbors, explaining the progressive suppression of the *o* phase and the emergence of *r*-phase dominance under nanoscale confinement. Correspondingly, *r*-phase–dominant HZO capacitors exhibit low coercive fields (*E*_coercive_ = 0.65 MV cm^–1^) and enhanced dielectric permittivity (*ε*_*r*_ ≈ 39.1), in close agreement with DFT trends. Notably, these values rival the performance of Hf­(Zr)-rich intercalated Hf­(Zr)_1+*x*_O_2_ (*x* = 0.079; *E*_coercive_ = 0.65 MV cm^–1^, *ε*_*r*_ = 33.15) reported previously,^4^ despite the complete absence of intercalation in the present system.

Collectively, these results establish a symmetry-governed pathway toward scalable, low-voltage ferroelectricity that transcends the conventional orthorhombic paradigm. The combination of intrinsically soft polarization switching and enhanced dielectric responsiveness in *R3*-HZO provides a compelling platform for ultralow-power nonvolatile memories, ferroelectric logic, and neuromorphic computing architectures, positioning symmetry engineering as a foundational strategy for next-generation ferroic materials.

## Supplementary Material



## Notes

Additional details on structural characterization and computational procedures are provided, including the construction and validation of the proposed *R3* model; the DFT+*U* calculation parameters; the resulting band structure and projected density of states analyses; the NEB calculations and the energy landscape results; and experimental fabrication and measurement protocols (PDF)
