# Supplementary material for: Symmetry-Guided Functional Pathways of Intercalation-Free Rhombohedral (R3) Hafnia Derived from the Fluorite Phase for Low-Coercive Ferroelectric Memory
Source: ACS Appl Mater Interfaces. 2026 Apr 1;18(14):20624–34. doi: 10.1021/acsami.5c23427 (PMC13088028; doi:10.1021/acsami.5c23427)
Supplement: Supplementary file 1 [file am5c23427_si_001.pdf]

Supporting Information

Symmetry-Guided Functional Pathways of Intercalation-Free Rhombohedral (*R3*) Hafnia Derived from the Fluorite Phase for Low-Coercive Ferroelectric Memory

Mochamad Januar[1*], Cheng-Hong Liu[1], Abhijit Aich[2], Jia-Yang Lee[1], Siddheswar Maikap[2,3], Min-Hung Lee[1,4,5*]

[1]Program for Semiconductor Devices, Materials, and Hetero-integration, Graduate School of Advanced Technology, National Taiwan University, Taipei, 106319, Taiwan.
[2]Thin Film Nano Tech. Lab., Department of Electronics Engineering, Chang Gung University, Taoyuan, 33302, Taiwan.
[3]Department of Obstetrics and Gynecology, Keelung Chang Gung Memorial Hospital, Keelung, 204, Taiwan.
[4]Graduate Institute of Electronics Engineering, National Taiwan University, Taipei, 106319, Taiwan.
[5]Institute of Applied Mechanics, National Taiwan University, Taipei, 106319, Taiwan.

*Corresponding author(s). E-mail(s): mochjanuar@ntu.edu.tw; minhunglee@ntu.edu.tw;

S1 Electronic Structure of 9-Atom and 12-Atom *R3* HfO_2

Figures S1 and S2 present the DFT+U band structures and projected densities of states (PDOS) for the 9-atom and 12-atom rhombohedral (*R3*) configurations of HfO_2, respectively. To correct the well-known band-gap underestimation inherent to conventional DFT, on-site Hubbard corrections of $U_{\mathrm{Hf-5d}} = 6.0$ eV and $U_{\mathrm{O-2p}} = 4.0$ eV were applied. In both configurations, the electronic structure exhibits wide-gap insulating behavior, with the valence band maximum (VBM) dominated by O 2p states and the conduction band minimum (CBM) primarily composed of Hf 5d orbitals—features characteristic of a fluorite-derived oxide lattice.

The 9-atom primitive cell yields a direct band gap of approximately 4.1 eV at the Γ point, while the 12-atom configuration exhibits a widened gap of about 6.2 eV. This increase arises from complete lattice relaxation and the inclusion of long-range polar distortion, which suppress band-edge tailing and enhance orbital localization. The broader gap in the 12-atom model thus reflects the electronic stabilization associated with symmetry breaking and polarization ordering within the rhombohedral network. Collectively, these results confirm that both structural representations capture the intrinsic electronic topology of rhombohedral HfO_2, whereas the 12-atom configuration more accurately reproduces the anisotropic band-edge renormalization and polarization-induced reconstruction intrinsic to the *R3* phase.

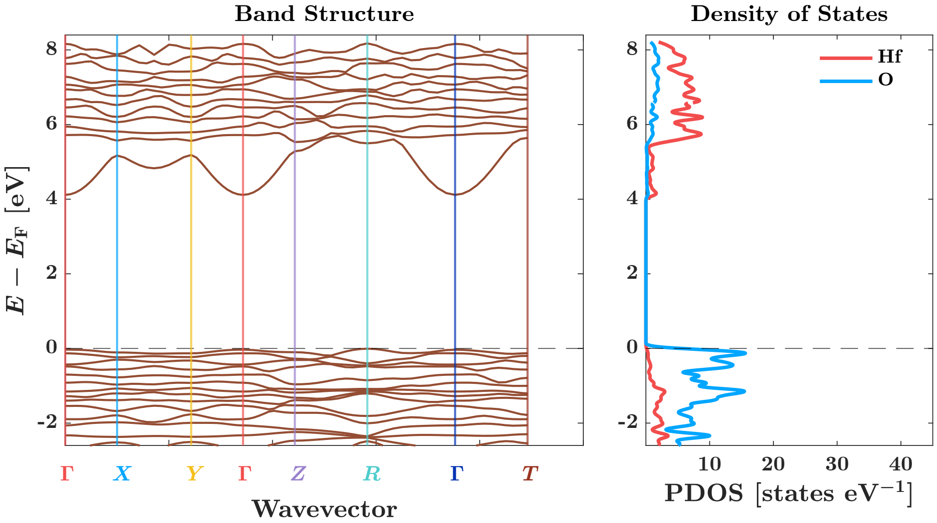

Fig. S1: DFT + U band structure (left panel) and PDOS (right panel) of 9-atom *R3*-HfO_2. The calculation shows a smaller insulating gap between O 2p valence and Hf 5d conduction states.

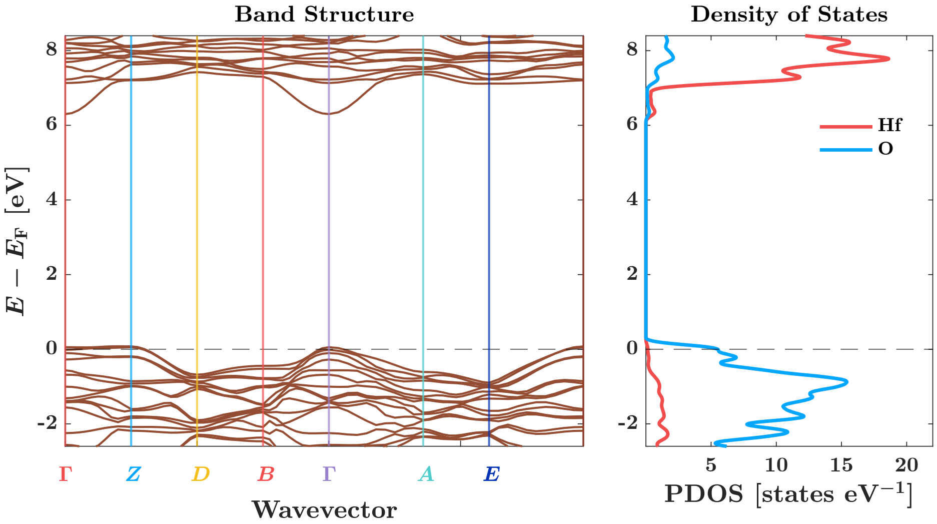

Fig. S2: DFT + U band structure (left panel) and PDOS (right panel) of 12-atom *R3* HfO_2. The calculation shows a larger insulating gap between O 2p valence and Hf 5d conduction states.

S2 Construction and Validation of the Fluorite-Derived Rhombohedral Model

S2.1 From monoclinic $P2_1/c$ to a symmetry-consistent rhombohedral $R3$ reference

The rhombohedral structural model employed in this work was systematically derived from the relaxed monoclinic $P2_1/c$ ground state of hafnia. After a full DFT+U relaxation of the monoclinic cell, the atomic coordinates were retained while the lattice metric was symmetrized by constraining the interaxial angles to $\alpha = \beta = \gamma = 88.71°$. This procedure maps the relaxed monoclinic ground state onto a centrosymmetric, non-polar rhombohedral frame, thereby providing a well-defined reference within the $R3$ symmetry group. Subsequent relaxation within this symmetry permits spontaneous symmetry breaking into a polar variant, enabling direct energy comparison ($\Delta E_{\text{centro-polar}}$) between non-polar and polar states.

S2.2 Computational parameters based on convergence tests

All calculations were performed using the Quantum ESPRESSO package with DFT+U in the orthogonalized-atomic scheme. The pseudopotentials were drawn from the PSLibrary (PAW, PBE-GGA). The convergence test for the plane-wave energy cutoff (E_{cut}) and k-point mesh was carried out to determine optimal settings that ensure reliable total energy predictions while minimizing computational cost. Figure S3 summarizes the convergence test results.

For the k-point mesh, a range of Monkhorst-Pack grids from $n = 2$ to $n = 8$ with and without offset ($s = 0$ and $s = 1$) was explored. The most stable region corresponds to a plateau in total energy differences (ΔE). The best compromise between accuracy and efficiency was achieved with a $5 \times 5 \times 5$ grid and no offset ($n = 5$, $s = 0$), yielding a minimum ΔE of 0.0 meV and a wall time of 436.87 s.

For the energy cutoff test, values from 35 Ry to 77 Ry were tested. While convergence in total energy differences is gradual, a plateau appears starting from 71 Ry. At this point, $\Delta E = 23.258$ meV, and further increases yield diminishing returns. Therefore, $E_{\text{cut}} = 71$ Ry was selected as the optimal cutoff, balancing precision and performance with a wall time of 680.77 s. These optimized parameters—$E_{\text{cut}} = 71$ Ry and $5 \times 5 \times 5$ k-points—are used for all subsequent DFT calculations to ensure numerical accuracy. Therefore, the convergence tested main computational parameters are summarized in Table S1.

Table S1: DFT+U parameters used for both monoclinic $P2_1/c$ and rhombohedral $R3$ calculations. Identical settings were applied to ensure consistency in total-energy comparisons.

**Parameter**	**Value**
Plane-wave cutoff (`ecutwfc`)	71 Ry (∼966 eV)
Charge-density cutoff (`ecutrho`)	639 Ry
Brillouin zone sampling	$5 \times 5 \times 5$ Monkhorst–Pack, shifted (1,1,1)
Occupations	Fixed (insulating)
Number of bands (`nbnd`)	72
Mixing mode	Plain
Mixing β	0.7
SCF convergence threshold	1.0×10^{-8} Ry
Spin treatment	Non-magnetic ($n_{\text{spin}} = 1$)
Hubbard U corrections	Hf-5d: 6.0 eV; Zr-4d: 6.0 eV; O-2p: 4.0 eV
Number of atoms per cell	12 (4 cations + 8 O)

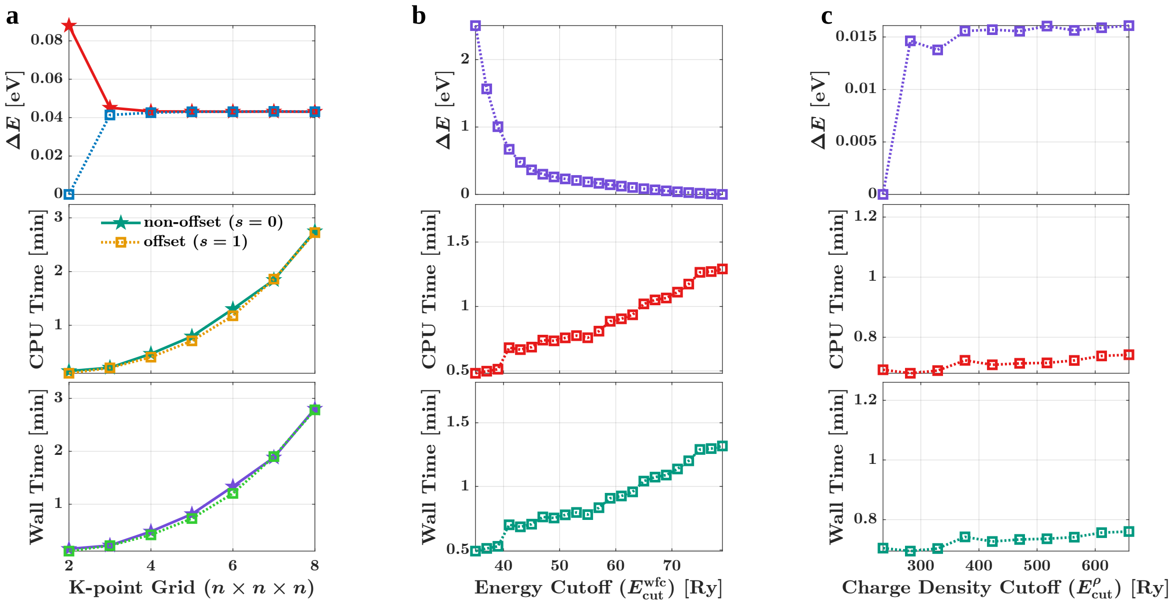

Fig. S3: **Convergence analysis of the total energy difference ΔE [eV] with respect to key simulation parameters.** **a**, ΔE as a function of $n \times n \times n$ Monkhorst-Pack k-point grids, comparing offset ($s = 1$) and no-offset ($s = 0$) schemes. CPU Time and Wall Time are also plotted to assess computational cost. **b**, ΔE, CPU Time, and Wall Time as functions of plane-wave energy cutoff $E_{\text{cut}}^{\text{wfc}}$ [Ry]. **c**, ΔE, CPU Time, and Wall Time as functions of charge density cutoff E_{cut}^{ρ} [Ry]. Plateau regions in each plot indicate optimal parameter choices balancing accuracy and efficiency.

S2.3 Rhombohedral cell vectors

The rhombohedral cell obtained after angle symmetrization has the following lattice vectors:

$$\mathbf{a}_1 = (5.2985,\ 0.0000,\ 0.0000)\ \text{Å}, \quad \mathbf{a}_2 = (0.1193,\ 5.2971,\ 0.0000)\ \text{Å}, \quad \mathbf{a}_3 = (0.1193,\ 0.1166,\ 5.2958)\ \text{Å}.$$

These correspond to a nearly cubic fluorite lattice slightly distorted into rhombohedral form, consistent with $\alpha = \beta = \gamma = 88.71°$. This structure serves as the centrosymmetric reference for assessing intrinsic polar instabilities.

S2.4 Wyckoff Positions of the Polar Rhombohedral *R3* Phase

The atomic configuration of the proposed rhombohedral ferroelectric phase of $Hf_{1-x}Zr_xO_2$ conforms to the *R3* space group (No. 146), which belongs to the trigonal crystal system with a rhombohedral Bravais lattice. In its hexagonal representation, the atoms occupy symmetry-allowed Wyckoff positions that enable a polar distortion along the $\langle 111 \rangle$ axis, thereby breaking inversion symmetry relative to the centrosymmetric counterpart.

- **Hf/Zr cations** occupy the general $6a$ Wyckoff position:

$$(x, y, z), \quad (-y, x-y, z), \quad (-x+y, -x, z),$$

$$(-x, -y, -z), \quad (y, -x+y, -z), \quad (x-y, x, -z),$$

  where (x, y, z) reflects the compositional mixing of Hf and Zr. The $6a$ site in *R3* allows displacements along the polar axis, giving rise to spontaneous polarization.
- **Oxygen anions** are distributed over the general $18b$ Wyckoff site:

$$(x, y, z), \quad (-y, x-y, z), \quad (-x+y, -x, z), \ldots$$

  which generates 18 equivalent positions through threefold rotational and inversion-breaking symmetry operations. Small axial displacements of these oxygens relative to the cation sublattice are

responsible for the polar distortion that stabilizes the ferroelectric state. In practice, representative values of (x, y, z) deviate only slightly from the ideal fluorite positions, but the collective displacement pattern establishes a net polarization along the rhombohedral c axis.

This atomic configuration corresponds to a 12-atom primitive cell (4 cations and 8 anions), directly comparable to the conventional fluorite lattice. The critical distinction lies in the *R3* symmetry, which allows symmetry-breaking polar displacements that reduce the total energy relative to the centrosymmetric reference derived from the monoclinic phase, thereby providing an intrinsic route to ferroelectricity in intercalation-free $Hf_{1-x}Zr_xO_2$. This structural framework establishes the basis for our first-principles investigation of composition-dependent stability, switching barriers, and coercive-field behavior in the rhombohedral phase.

S2.5 Structural Optimization

To illustrate the construction and evaluation of hafnia polymorphs, we examined two representative configurations: the monoclinic $P2_1/c$ phase, serving as the initial ground-state structure, and the rhombohedral *R3* phase, representing the targeted high-symmetry configuration. First-principles equation-of-state (EOS) fittings were performed for both to determine their equilibrium volumes, bulk moduli, and total energies, enabling direct comparison of their relative stability and compressibility.

For the anisotropic monoclinic $P2_1/c$ phase, the equilibrium geometry was determined through a sequential optimization of the lattice constants a, b, and the c/a ratio. Each parameter was varied independently while fixing the others to the values optimized in preceding steps, ensuring convergence toward the global energy minimum. The total energy–volume data from each sweep were fitted using four standard EOS models—Murnaghan, Birch, Birch–Murnaghan, and Vinet—to determine the equilibrium volume (V_0), bulk modulus (B_0), minimum energy (E_0), pressure derivative (B'), and optimized lattice parameters. The combined results (Table S2) show that all sweeps converge to consistent equilibrium conditions of $V_0 = 144.95$ Å^3, $a_{\mathrm{eq}} = 5.213$ Å, and $c/a \approx 1.032$, confirming the internal consistency of the optimization. Among the models, the Birch–Murnaghan fit yields the lowest total energy ($E_0 = -42417.11$ eV) and the highest bulk modulus ($B_0 = 431.7$ GPa), reaffirming the dense and mechanically stiff character of the monoclinic phase.

Table S2: Combined equation-of-state (EOS) fitting results for the monoclinic $P2_1/c$ HfO_2 structure. Values correspond to the fully optimized configuration following sequential a-, b-, and c/a-sweeps.

**Model**	V_0 **[Å^3]**	B_0 **[GPa]**	E_0 **[eV]**	B'	a_{eq} **[Å]**	b_{eq} **[Å]**	c/a_{eq}
Murnaghan	144.95	389.08	-42416.9302	4.19	5.213	5.213	1.032
Birch	144.95	399.24	-42416.9716	4.13	5.213	5.213	1.032
Birch-Murnaghan	144.95	431.71	-42417.1100	0.59	5.213	5.213	1.032
Vinet	144.95	403.57	-42416.9892	4.11	5.213	5.213	1.032

In contrast, the rhombohedral *R3* phase—with $a = b = c$ and equal rhombohedral angles—required only a single isotropic volume sweep. The EOS fits summarized in Table S3 yield $V_0 = 146.99$ Å^3, $a_{\mathrm{eq}} = 5.298$ Å, and $B_0 \approx 200$–290 GPa, depending on the fitting model. The Birch–Murnaghan model again provides the best description, giving $E_0 = -42417.27$ eV and $B_0 = 288$ GPa, indicative of a more compressible and elastically compliant lattice relative to the monoclinic reference.

Table S3: Equation-of-state (EOS) fitting results for the rhombohedral *R3* HfO_2 structure. The lower bulk modulus and larger equilibrium volume signify enhanced compressibility relative to the monoclinic phase.

**Model**	V_0 **[Å^3]**	B_0 **[GPa]**	E_0 **[eV]**	B'	a_{eq} **[Å]**	c/a_{eq}
Murnaghan	146.99	198.89	-42416.4593	3.58	5.298	1.000
Birch	146.99	207.90	-42416.5121	3.98	5.298	1.000
Birch-Murnaghan	146.99	288.29	-42417.2721	0.01	5.298	1.000
Vinet	146.99	213.05	-42416.5444	4.17	5.298	1.000

Overall, the monoclinic $P2_1/c$ phase exhibits a denser and stiffer lattice with $B_0 \approx 400$–430 GPa, confirming its role as the thermodynamic ground state. The rhombohedral *R3* phase, while possessing

a larger equilibrium volume and significantly lower bulk modulus ($B_0 \approx 260$ GPa), remains energetically competitive within approximately 0.1–0.2 eV per 12-atom cell. The enhanced elastic compliance of the *R3* structure reflects its capacity to accommodate symmetry-lowering distortions, providing a favorable precursor framework for the emergence of polar order in doped or strained hafnia-based ferroelectrics.

S2.6 Atomic Position Relaxation

Building upon the optimized lattice parameters obtained from the EOS analysis, the atomic positions of rhombohedral $Hf_{0.5}Zr_{0.5}O_2$ were further relaxed using the `relax` calculation in Quantum ESPRESSO. This step refines the internal atomic coordinates while maintaining the equilibrium cell dimensions fixed in the structural optimization. The relaxation resulted in moderate adjustments, including a slight in-plane lattice expansion (a and b) and a minor contraction along the c-axis, leading to an overall volume increase of approximately 2.1%. The largest displacements were observed among oxygen atoms, reflecting local symmetry-preserving distortions that accommodate internal strain relaxation. Importantly, the refined fractional coordinates and lattice parameters remain consistent with the *R3* symmetry in its hexagonal representation, confirming the robustness of the rhombohedral framework obtained from the EOS fitting.

S2.7 Crystallographic Summary

Table S4: Crystallographic summary of the polar rhombohedral $Hf_{1-x}Zr_xO_2$ (*R3*) structure employed in this work.

**Crystal System**	Trigonal
**Bravais Lattice**	Rhombohedral (hexagonal representation)
**Space Group**	*R3* (No. 146, polar)
**Lattice Representation**	Hexagonal
**Lattice Constants**	$a = 5.298$ Å, $b = 5.297$ Å, $c = 5.296$ Å
**Interaxial Angles**	$\alpha = \beta = \gamma = 88.71^\circ$
**Atoms per Primitive Cell**	12 (4 Hf/Zr and 8 O)
**Wyckoff Positions**	$6a$ for Hf/Zr, $18b$ for O

S2.8 Symmetry Verification

To validate the structural model, we employed `spglib` and the internal symmetry analysis module of QUANTUM ESPRESSO. Both consistently identified the structure as belonging to the *R3* space group (No. 146), with trigonal (rhombohedral) symmetry. The loss of inversion symmetry relative to the parent non-polar reference confirms the polar distortion along the $\langle 111 \rangle$ axis, consistent with the emergence of spontaneous polarization.

This polar rhombohedral structural framework thus provides a symmetry-consistent and computationally robust foundation for exploring intrinsic stabilization, polarization switching, and electronic properties across varying Hf:Zr ratios in $Hf_{1-x}Zr_xO_2$.

S3 Computational Workflow for Non-Polar–Polar Phase Comparison

To investigate the intrinsic stabilization of ferroelectricity in rhombohedral $Hf_{1-x}Zr_xO_2$, we conducted a systematic first-principles comparison between centrosymmetric non-polar and symmetry-broken polar states within the *R3* lattice. Both configurations were constructed at an equiatomic Hf:Zr ratio (1:1) and contained 12 atoms per primitive cell, ensuring direct comparability without the influence of dopants, strain, or intercalation. Each structure was fully relaxed under identical convergence criteria, plane-wave cutoffs, k-point grids, and pseudopotentials to guarantee a consistent energy reference.

To further characterize the ferroelectric response, the spontaneous polarization of both configurations was evaluated using the modern theory of polarization based on the Berry-phase formalism. Self-consistent field calculations were performed with `lberry = .true.` and dense k-point sampling. As expected, the centrosymmetric reference yielded $P_s \approx 0$, whereas the symmetry-broken structure exhibited a finite spontaneous polarization oriented along the $\langle 111 \rangle$ axis. Polarization values were obtained in atomic units and converted to μC cm^{-2} for comparison with experiment.

The kinetic accessibility of the polar phase was assessed through nudged elastic band (NEB) calculations connecting the non-polar and polar states. Intermediate images were generated by linear interpolation between the two relaxed structures, and a chain of 5–9 images was optimized using the climbing-image NEB method. The resulting double-well energy profile provided both the switching barrier height and the effective coercive field associated with the non-polar–polar transition.

This unified workflow establishes a consistent framework for disentangling the intrinsic stabilization of ferroelectricity in $R3$-$Hf_{1-x}Zr_xO_2$. By contrasting centrosymmetric and symmetry-broken states within the same lattice, it reveals the thermodynamic preference, polarization magnitude, and transition barrier of the rhombohedral phase without invoking extrinsic mechanisms—highlighting the purely symmetry-driven origin of ferroelectricity in this intercalation-free material system.

S4 Role of the Tetragonal Phase as an Intermediate in the Polar Switching Landscape

To explicitly assess the possible involvement of the tetragonal phase in the polarization switching pathway of $Hf_{0.5}Zr_{0.5}O_2$, we performed NEB calculations connecting the orthorhombic $Pca2_1$ phase and the rhombohedral $R3$ phase, with the tetragonal $P4_2/nmc$ structure employed as the central reference configuration. This approach allows a direct evaluation of whether the tetragonal phase can act as an intermediate state linking competing polar polymorphs through continuous symmetry-breaking distortions.

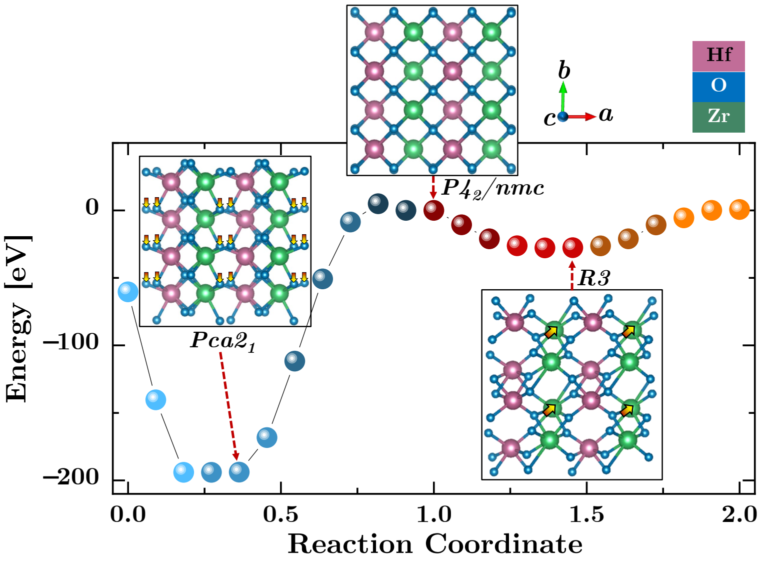

Fig. S4: **NEB-calculated energy profile along a structural reaction coordinate linking the $Pca2_1$, $P4_2/nmc$, and $R3$ phases of $Hf_{0.5}Zr_{0.5}O_2$.** The tetragonal $P4_2/nmc$ phase occupies an intermediate position between two symmetry-broken polar minima. Insets show representative atomic configurations, and arrows indicate the directions of polar distortion.

Figure S4 shows the resulting minimum-energy path constructed between the $Pca2_1$ and $R3$ phases. The tetragonal $P4_2/nmc$ structure occupies a saddle-like position along the reaction coordinate, separating two symmetry-broken polar minima. From this high-symmetry reference, the system can evolve toward the lower-energy $Pca2_1$ phase on one side or toward the slightly higher-energy $R3$ phase on the other. The relative depth of the two minima confirms that the orthorhombic phase remains thermodynamically more stable than the rhombohedral phase at this composition, consistent with prior reports.

Importantly, these results demonstrate that both polar phases are accessible from the tetragonal configuration through continuous lattice distortions, establishing the $P4_2/nmc$ phase as a natural intermediate or transition reference in the switching landscape. Within this framework, a direct $Pca2_1 \leftrightarrow R3$ transition is not assumed; instead, polarization reversal proceeds via branching pathways that originate from the tetragonal state and relax toward different polar symmetries.

This intermediate role of the tetragonal phase also provides a microscopic basis for the experimentally observed non-ideal squareness of the P–V hysteresis loops in polycrystalline devices.

The coexistence and transient participation of tetragonal and orthorhombic regions during electrical cycling can lead to distributed switching fields and incomplete polarization saturation, even in r-phase–dominant films.

S5 Experimental Methods

S5.1 Device Fabrication

The metal–ferroelectric–metal (MFM) capacitor structure, schematically illustrated in Fig. S5, was fabricated on a 6-inch n^+–Si substrate. A 30 nm TiN bottom electrode was deposited by physical vapor deposition (PVD), followed by a 10 nm $Hf_{0.5}Zr_{0.5}O_2$ (HZO) layer grown by atomic layer deposition (ALD). To investigate the effect of interfacial chemistry, two sample sets were prepared—with and without H_2 plasma surface treatment prior to top electrode deposition. Subsequently, a 50 nm TiN top electrode was deposited by PVD and patterned lithographically to define the capacitor geometry. Crystallization of the HZO layer was achieved by rapid thermal annealing (RTA) at 500 °C for 60 s in a nitrogen ambient, resulting in high-quality TiN/HZO/TiN stacks suitable for evaluating rhombohedral-phase ferroelectric behavior.

For comparison, orthorhombic-dominant HZO capacitors fabricated under standard conditions were referenced from previously published work.[1] These samples served as a control set for benchmarking structural and electrical characteristics against the rhombohedral-phase–dominant devices.

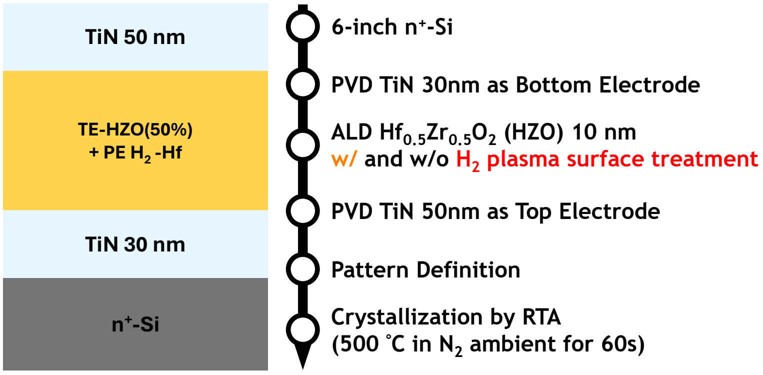

Fig. S5: Device structure (left) and fabrication flow (right) of TiN/$Hf_{0.5}Zr_{0.5}O_2$/TiN capacitors.

S5.2 Electrical Characterization

The coercive field (E_{coercive}) was extracted from capacitance–voltage (C–V) measurements as the electric field corresponding to the capacitance maximum during bipolar voltage sweeps. The reported E_{coercive} values represent the average of the positive and negative switching fields, following established procedures in the literature.[2] This definition enables a consistent comparison between electrical switching behavior and the crystallographic phase content determined by microscopy- and diffraction-based analyses.

To rigorously separate ferroelectric switching from non-switching and leakage contributions, pulsed polarization measurements were carried out using the standard PUND (positive-up–negative-down) protocol. Pulse widths of approximately 1–10 μs and pulse amplitudes matched to those used in the corresponding P–V measurements were employed. Representative PUND-extracted hysteresis loops measured before and after electrical conditioning are shown in Fig. S6. All electrical characterizations were conducted at room temperature using identical pad-contact probing conditions to ensure measurement consistency across devices.

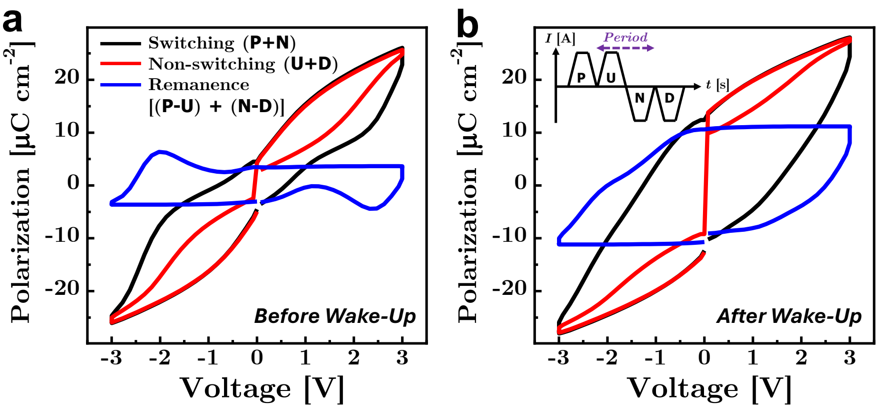

Fig. S6: PUND-extracted polarization hysteresis loops measured **(a)** before and **(b)** after electrical conditioning, showing enhanced ferroelectric switching, increased remanent polarization, and improved loop symmetry after wake-up cycling.

Direct-current current–voltage (DC I–V), sweep-rate–dependent I–V, and polarization–voltage (P–V) measurements were subsequently performed using triangular voltage waveforms. Sweep times of 100 ms, 10 ms, 1 ms, and 0.1 ms were employed to probe conduction and switching dynamics over a wide range of effective measurement rates. Representative sweep-rate–dependent I–V characteristics for o-phase– and r-phase–dominant capacitors are shown in Fig. S7. The maximum applied electric field was limited to approximately ±3–4 MV cm^{-1}, remaining below the dielectric breakdown threshold for all devices.

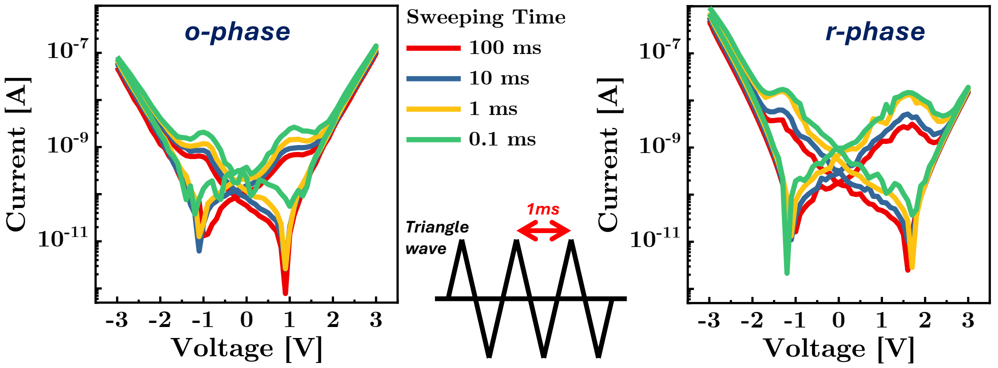

Fig. S7: Sweep-rate–dependent I–V characteristics of o-phase–dominant and r-phase–dominant HZO capacitors.

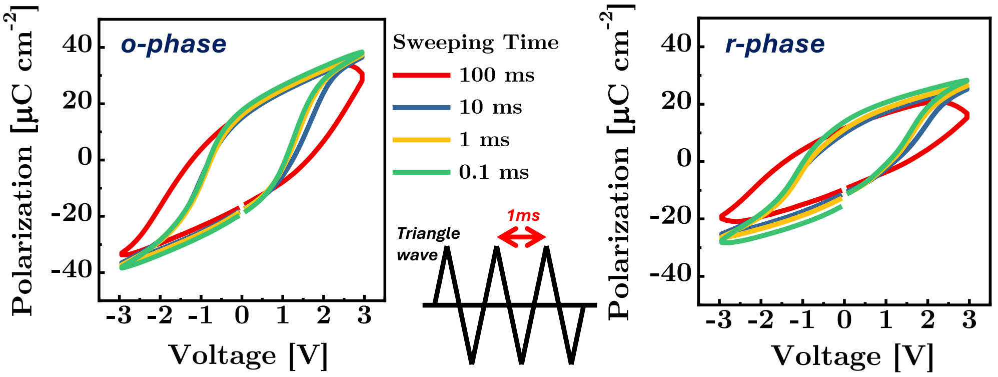

Fig. S8: Sweep-rate–dependent P–V characteristics of o-phase–dominant and r-phase–dominant HZO capacitors.

Quasi-static P–V hysteresis loops were measured at frequencies between 1 and 10 kHz using triangular waveforms, as illustrated in the sweep-rate–dependent P–V data in Fig. S8. Electrical wake-up cycling was applied prior to post-conditioning measurements to activate pre-existing ferroelectric domains and stabilize the switching response.

References

[1] Hsiang, K.-Y. *et al.* Correlation between access polarization and high endurance ($\sim 10^{12}$ cycling) of ferroelectric and anti-ferroelectric $HfZrO_2$. *2022 IEEE International Reliability Physics Symposium (IRPS)* P9–1–P9–4 (2022).

[2] Mukherjee, S. *et al.* Resolving the discrepancy between coercive voltages extracted from C-V and P-V measurements in a ferroelectric capacitor. *Solid-State Electronics* **212**, 108834 (2024).
